# The “Ants of Cyprus” website: a dynamic, online awareness raising and conservation tool

**DOI:** 10.3897/BDJ.13.e141679

**Published:** 2025-01-28

**Authors:** Jakovos Demetriou, Christos Georgiadis, Sebastian Salata, Lech Borowiec, Mathias Dillen, Quentin Groom, Evan P. Economo, Helen E. Roy, Angeliki F. Martinou

**Affiliations:** 1 Laboratory of Vector Ecology and Applied Entomology, Joint Services Health Unit Cyprus, BFC RAF Akrotiri, Limassol, Cyprus Laboratory of Vector Ecology and Applied Entomology, Joint Services Health Unit Cyprus, BFC RAF Akrotiri Limassol Cyprus; 2 Enalia Physis Environmental Research Centre, Acropoleos 2, Aglantzia 2101, Nicosia, Cyprus Enalia Physis Environmental Research Centre, Acropoleos 2, Aglantzia 2101 Nicosia Cyprus; 3 Department of Ecology and Systematics, Faculty of Biology, National and Kapodistrian University of Athens, Athens, Greece Department of Ecology and Systematics, Faculty of Biology, National and Kapodistrian University of Athens Athens Greece; 4 Section of Zoology-Marine Biology, Faculty of Biology, National and Kapodistrian University of Athens, Athens, Greece Section of Zoology-Marine Biology, Faculty of Biology, National and Kapodistrian University of Athens Athens Greece; 5 Zoological Museum of the University of Athens, Athens, Greece Zoological Museum of the University of Athens Athens Greece; 6 University of Wroclaw, Department of Biodiversity and Evolutionary Taxonomy, Myrmecological Laboratory, Wroclaw, Poland University of Wroclaw, Department of Biodiversity and Evolutionary Taxonomy, Myrmecological Laboratory Wroclaw Poland; 7 Meise Botanic Garden, Meise, Belgium Meise Botanic Garden Meise Belgium; 8 Okinawa Institute of Science and Technology, Kunigamigun, Okinawa, Japan Okinawa Institute of Science and Technology Kunigamigun, Okinawa Japan; 9 UK Centre for Ecology and Hydrology, Oxfordshire, United Kingdom UK Centre for Ecology and Hydrology Oxfordshire United Kingdom; 10 Department of Ecology and Conservation, University of Exeter, Exeter, United Kingdom Department of Ecology and Conservation, University of Exeter Exeter United Kingdom; 11 Climate and Atmosphere Research Centre/Care-C, The Cyprus Institute, Athalassa Campus, Nicosia, Cyprus Climate and Atmosphere Research Centre/Care-C, The Cyprus Institute, Athalassa Campus Nicosia Cyprus

**Keywords:** biodiversity, community science, Formicidae, Google sites, online resources, website

## Abstract

Ants are an important arthropod group due to their involvement in ecological processes amongst others as ecosystem engineers or predators, but some invasive alien species are also implicated in detrimental environmental, economic and human health effects. Despite recent advancements, the ant biodiversity of Cyprus is still in need of further research with previous online species inventories synthesising a checklist of just 65 native and 10 alien species. The “Ants of Cyprus” website (https://sites.google.com/view/ants-of-cyprus) aims to: (1) raise public awareness and increase local knowledge on the biodiversity and ecological significance of ants, (2) provide ecological data and (3) enhance conservation efforts and management practices for native and alien ant species, respectively. This dynamic, user-friendly website has been designed for use by the scientific community, conservation officers and the wider public and it will be regularly updated as new information and data are generated. Additionally, this information will be critical for reporting on progress towards biodiversity targets and goals.

## Introduction

Access to entomological knowledge and stronger engagement with the public are amongst the emerging themes and future priority challenges to entomology (Luke et al. 2023). Biodiversity data platforms, repositories, fora, databases and websites contribute to these challenges by raising awareness, increasing local knowledge of biodiversity and promoting public participation in scientific research (McCormack 2007, Borges et al. 2010, Goula et al. 2013, Kalaentzis et al. 2021, Zotos et al. 2023). Furthermore, biodiversity databases have been extensively used for mapping and modelling species distributions, reporting on newly-detected or expanding alien species, as well as in studies on the ecology and conservation of rare or threatened species (Castañeda et al. 2019, Mesibov 2019, Avtaeva et al. 2020, Ruzzier et al. 2020, Wilson et al. 2020, Davranoglou and Karaouzas 2021, Angelidou et al. 2022, Krapf 2023, Lugo et al. 2023, Skvarla and Fisher 2023). Despite their high biodiversity, insects are amongst the classes with the lowest median observations per species and poorest taxonomic precision in the Global Biodiversity Information Facility (GBIF) (Troudet et al. 2017). This taxonomic bias is also evident in global conservation efforts, with insect conservation receiving minimal attention compared to vertebrates (Dunn 2005, Oberhauser and Guiney 2009, Donaldson et al. 2017).

Recently, large scale data syntheses have developed taxonomic resources for an exemplar invertebrate group, ants (Janicki et al. 2016, Guénard et al. 2017, Kass et al. 2022). Ants (Hymenoptera, Formicidae) belong to an ecologically important family of insects, with recent global estimates showing 20 quadrillion ants on Earth and their total global biomass reaching 12 megatons of dry carbon (Parker and Kronauer 2021, Schultheiss et al. 2022). They are considered “ecosystem engineers”, altering ecosystem dynamics and the availability of resources to other species directly or indirectly through the creation of their nests (Jones et al. 1997, Jouquet et al. 2006, Del Toro et al. 2012). Ants also form symbiotic relationships with animals (myrmecophiles), plants and fungi, with the latter relationship increasing soil biodiversity through mycorrhizal enrichment, dispersion of fungal spores, the development of plant rhizosphere fauna and enhancing the growth of ammonifying bacteria (Folgarait 1998). The nests and tunnels which ants excavate also increase soil drainage and aeration, making water and air more accessible to soil arthropods and plant roots (Folgarait 1998). Their movement between shallow and deeper soil layers transports faeces, corpses and other organic matter, enhancing their availability to decomposers and, thus, enriching the soil with nutrients (Folgarait 1998, Del Toro et al. 2012, De Almeida et al. 2020). Regarding ant-plant interactions, myrmecochory, i.e. the dispersal of seeds by ants, plays an important role for at least 11,000 species of plants (Lengyel et al. 2010). Some ants have also been found to pollinate plants such as orchids (Del Toro et al. 2012, Claessens and Seifert 2017). In addition to their aforementioned roles in ecosystem processes, ants have direct benefits for people both as a food source and, more recently, in pharmaceutical and biomedical applications which use their silk cocoons or venom (Del Toro et al. 2012).

On the other hand, some ant species are regarded as agricultural, commercial and household pests, often being reported as a nuisance for inflicting painful stings or bites and infesting food products, electrical appliances or timber (Klotz et al. 1995, Lee 2002, Wetterer 2009, Del Toro et al. 2012, Serge et al. 2019). These “disservices” impact human and animal health, cause substantial economic losses across the globe and are exacerbated by the impact of *Linepithemahumile* (Mayr, 1868), *Solenopsisinvicta* Buren, 1972, *Wasmanniaauropunctata* (Roger, 1863) and other invasive alien species of ants. These invasive ants have affected ecosystems and their native biodiversity, leading, amongst others, to the decrease of native arthropod and vertebrate abundances (Wetterer and Porter 2003, Moreira et al. 2005, Lard et al. 2006, Del Toro et al. 2012, Zina et al. 2020, Gruber et al. 2022, Montgomery et al. 2022, Tercel et al. 2023).

Cyprus has been identified as a biodiversity hotspot (Myers et al. 2000) and important area for small-ranged ants (Kass et al. 2022). The myrmecofauna of Cyprus has been reported through online catalogues which include a checklist of only 65 native and ten alien species (Janicki et al. 2016, Guénard et al. 2017). Some information and annotations on selected species can be found in Georghiou (1977), Edwards et al. (2016), the Biodiversity of Cyprus blogspot (https://biodiversitycyprus.blogspot.com/search/label/Ants%20of%20%20Cyprus) and AntWiki (https://www.antwiki.org/wiki/Cyprus). However, recent research has added several both alien and native species to the inventory of Cyprus (Demetriou et al. 2023, Salata et al. 2023a, Salata et al. 2023b, Salata et al. 2023c, Salata et al. 2024). There are a number of websites and platforms focusing on particular taxonomic groups for Cyprus, such as BirdLife Cyprus (https://birdlifecyprus.org/), the Flora of Cyprus (Hand et al. 2011), the Cyprus Database of Alien Species (CyDAS) (https://ris-ky.info/cydas), the Cyprus Herp Atlas (Zotos et al. 2023), the Chalcid Wasps of Cyprus (Koutsoukos et al. 2024) and Biodiversity of Cyprus (https://biodiversitycyprus.blogspot.com/); however, a portal providing information and data on the ants of Cyprus is lacking.

**Figure 1.**
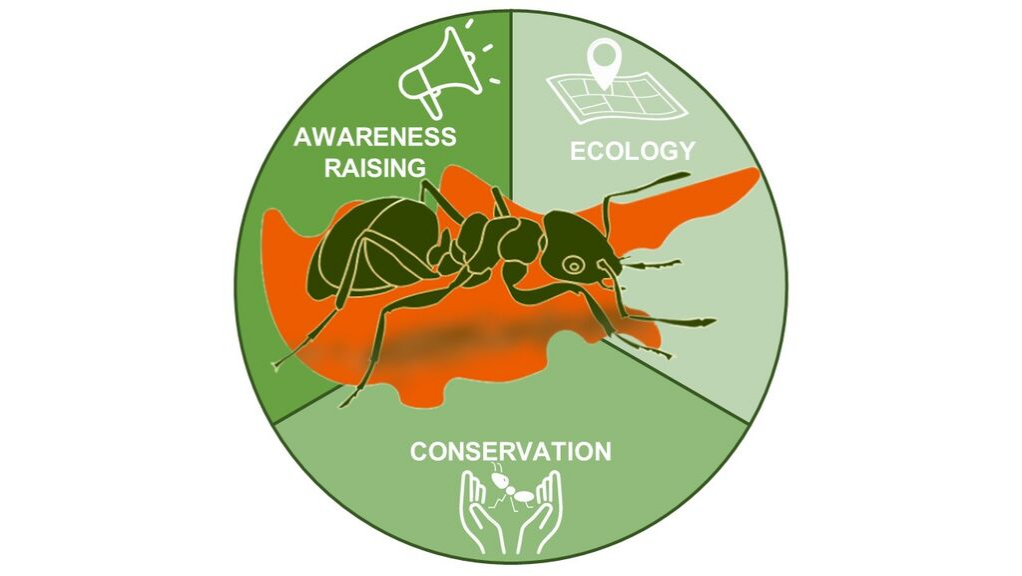
An illustration of the main objectives of the “Ants of Cyprus” website focusing on awareness raising, the ecology and conservation of ants on Cyprus.

The “Ants of Cyprus” website aims to aggregate, summarise and present all available knowledge on the ants of Cyprus. This dynamic, user-friendly website has been constructed to be used by the scientific community, conservation officials and the wider public. The website’s main objectives are:

(1) To raise public awareness and increase local knowledge of the biodiversity and ecological significance of ants on Cyprus and beyond.

(2) Provide data on the distribution (including downloadable occurrence records and distributional maps), phenology (monthly activity/presence), morphology (photographic material), habitats and interrelationships of ants with other insect species on Cyprus.

(3) Enhance conservation efforts and set a baseline for the future assessment of both native i.e. against IUCN categories and criteria and alien ant species i.e. using the Environmental Impact Classification for Alien Taxa (EICAT) and Socio‐Economic Impact Classification of Alien Taxa (SEICAT) protocols (Blackburn et al. 2014, Bacher et al. 2017), to provide information and guide national conservation and management priorities, respectively (Fig. 1).

## Materials and methods

### Construction of the website

An online account (ants.of.cyprus@gmail.com) was set up for the website and all photographic material, infographics, documents, sheets and data were stored in the provided Google Drive file storage. The “Ants of Cyprus” website (https://sites.google.com/view/ants-of-cyprus/home) was constructed on Google Sites as part of the DPLUS200 project “Biodiversity and spatiotemporal patterns of ants on Akrotiri Peninsula” (https://darwinplus.org.uk/project/DPLUS200/), funded by the Darwin Initiative – Biodiversity Challenge Funds. Infographics and stylised images were created on Canva graphic design platform (https://www.canva.com/en_gb/). The platform has been paired with Google Analytics to monitor website traffic.

### Construction of species profiles

Species taxonomy follows AntCat (Bolton 2024). The status of each species is assessed as either “native”, “native (endemic)”, “alien” or “alien (invasive)”. Species stated as “alien” represent non-native species introduced via human activities outside their native range (EU 1143/2014), whereas “invasive” are those alien species shown to adversely affect native biodiversity and, in some cases, people (Roy et al. 2024).

Photographs of specimens in species profiles were taken using a Nikon SMZ 1500 stereomicroscope, Nikon D5200 photo camera and Helicon Focus software. Whenever photographs were not available, we have used resources available online at AntWeb (2024). Usage of photographs for non-commercial purposes is permitted under proper attribution to source material, such as websites or scientific publications indicated in the “images” section on each species profile.

### Occurrence records

A literature review on the ant biodiversity of Cyprus was conducted, compiling references and distributional records of various species (e.g. Emery (1909), Emery (1910), Santschi (1930), Santschi (1934), Santschi (1937), Georghiou (1977), Buschinger and Douwes (1993), Seifert (2003), Beibl et al. (2007), Ionescu-Hirsch (2009), Seifert and Schultz (2009), Salata and Borowiec (2015a), Salata and Borowiec (2015b), Salata et al. (2019), Seifert (2020), Demetriou et al. (2022), Demetriou et al. (2023), Salata et al. (2023a), Salata et al. (2023b), Salata et al. (2023c), Salata et al. (2024)). Occurrence records were added to *.csv and *.xlsx files from available scientific literature. Where available, coordinates of occurrence records in distribution maps and *.xlsx files are given in decimal degrees (DD) in WGS84 coordinate system. Where literature records refer to specific localities, but no coordinates were provided, these have been added by the authors, followed by the annotation “coordinates not given in text”. Observations referring to “Cyprus” in general, without reference to specific localities were not accounted for during the compilation of distributional data files. For each species, a *.csv file including distributional data was imported to QGIS in order to create maps embedded in each species profile.

The Area of Occupancy (AOO) and Extent of Occurrence (EOO) for each species (where appropriate) were calculated via GeoCat (https://geocat.kew.org/editor) developed by IUCN (Bachman et al. 2011). The establishment status of each species is assessed as either: “Established” (established populations in the wild), “Indoors introduced” (without established populations in the wild, for example in buildings, greenhouses, airports, quarantine surveys) or “Unknown” (data-deficient species) (Demetriou et al. 2023). Establishment means of each species were recorded as per Darwin Core Maintenance Group (2021) with species assessed as: native, native: reintroduced, introduced, introduced: assisted colonisation, vagrant or uncertain. Habitat classification scheme follows IUCN (https://www.iucnredlist.org/resources/habitat-classification-scheme), whereas in species conservation, the IUCN Red List category follows IUCN (https://www.iucnredlist.org/). Threats classification scheme also follows IUCN (https://www.iucnredlist.org/resources/threat-classification-scheme) and presence in protected areas refer to the designated NATURA 2000 network sites on the island (https://natura2000.eea.europa.eu/) (92/43/EEC; 2009/147/EC) as well as the Akrotiri Peninsula RAMSAR site (site number: 1375) (https://rsis.ramsar.org/ris/1375).

The occurrence data were mapped to the Darwin Core standard (Wieczorek et al. 2012) and published to the Global Biodiversity Information Facility (GBIF) with dwc:basisOfRecord set as Material Citation (Demetriou et al. 2024). As more data are added to the website, this data publication to GBIF is also planned to be updated along with it. No voucher codes or other identifiers identifying the deposited specimens were available, but an attempt was made to at least have a persistent identifier for either the collection or institution at the location where the specimen was deposited at, to facilitate linking the citations to the published specimens in the future. For private collections, the ORCID of the owner was used as dwc:collectionID.

## Website overview

**Figure 2.**
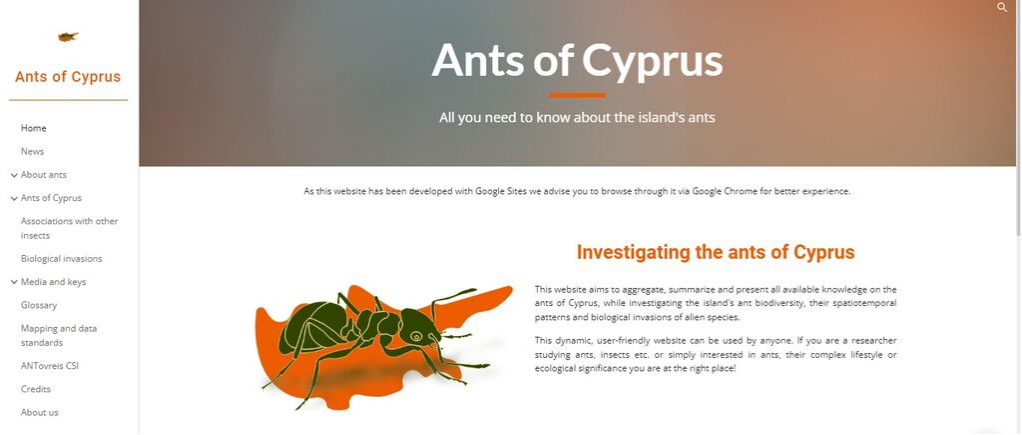
Ants of Cyprus homepage.

The main menu of the site is divided into the following top-level pages:

**Home.** Welcome page stating the funding declaration and the website’s main objectives (Fig. 2).

**News.** Blog page featuring the latest news on the ants of Cyprus, such as information on new publications, events or citizen-science initiatives taking place.

**About ants.** A menu section page further divided into the following pages: “How to spot an ant”, “Ants and other species”, “Ecological significance” and “Eusociality and lifecycle” providing valuable information on the morphology, species interrelationships and ecology of ants to the wider public.

**Ants of Cyprus.** A pull-down menu section in the form of a taxonomic backbone following Bolton (2024), with information on the subfamilies > genera > and species present on the island. On a subfamily level, the genera present in Cyprus are noted. Moving on to the genus level, an embedded map from the AntMaps website (Janicki et al. 2016, Guénard et al. 2017) shows the number of native species in the genus worldwide. The map is followed by a checklist of the species present on Cyprus, species previously reported, but dubious or erroneous (where applicable) and the relevant scientific literature. On a species level, profiles for all species found in Cyprus are provided including data on their status, distribution, phenology (monthly activity/presence), establishment status, ecology and habitats and conservation accompanied by photographic material, maps, downloadable occurrence records and relevant scientific literature.

**Associations with other insects.** This section provides an *.xlsx file on known associations of ants with other insects including myrmecophilus species and parasitoids, based on scientific literature. Myrmecophilous taxa, such as Brentidae, the genera *Cryptocephalus* (Coleoptera, Chrysomelidae), *Thorictus* (Coleoptera, Dermestidae) and lycaenid butterflies (Lepidoptera, Lycaenidae), whose host ants are unknown, have been also included in order to promote collaboration with and data collection from beetle and butterfly enthusiasts, ecologists, taxonomists and scientists. This file will be supplemented in the future with more species and associations as research on ants in Cyprus and associates develops.

**Biological invasions.** General information on biological invasions and the alien ants of Cyprus are provided, including clickable links to the species profiles of 17 alien ants currently detected on the island (Demetriou et al. 2023), with special reference to the presence and impacts of the little fire-ant *Wasmanniaauropunctata* (Roger, 1863) (Demetriou et al. 2022, Demetriou et al. 2023) as well as measures to mitigate further spread. A full disclosure statement is provided as the authors are not involved with pest control, clarifying the ecological nature of the website.

**Media and keys.** A menu section divided into the sub-pages “Statistics and checklist”, “Distributional data (published)”, “Educational material” and “Identification keys”. The “Statistics and checklist” subpage includes a dynamic checklist of ants on Cyprus alongside their status. At the top of the page, the number of subfamilies, genera and species present on the island is given, including the number and overall percentages of endemic and non-native species. The “Distributional data (published)” subpage includes a link to the published GBIF dataset (Demetriou et al. 2024), a .csv copy of the dataset, a map with all published data, as well as the citation to the dataset. The subsection “Educational material” includes a photographic guide to identify main ant groups, a trifold brochure on the ANTovreis citizen-science initiative, informational material on the little fire ant *Wasmanniaauropunctata* and posters on alien invasive species not yet reported from Cyprus, such as *Anoplolepisgracilipes*, *Linepithemahumile* and *Solenopsisinvicta*, all both in Greek and English. Links to research articles with identification keys, are provided in the appropriate section.

**Glossary.** Definition of entomological terms used throughout the website. A link to the “interpretative dictionary of entomological terms” in Greek by Buchelos (2012) is also provided to guide Greek-speaking readers.

**Mapping and data standards.** Listing materials and methods used for the construction of the species profiles as listed in the aforementioned section.

**ANTovreis CSI.** Information and encouragement to participate in a citizen-science scheme on Cypriot ants, which will be presented as the project evolves.

**Credits.** Acknowledging and listing data contributors and supporting organisations.

**About us.** Includes the website’s citation, project members and contact information (ants.of.cyprus@gmail.com).

## Data usage

### Awareness raising

Insects are commonly perceived as pests by the wider public. However, being involved in nature conservation from a young age, participating in insect-focused public activities or promoting insects in culture has been shown to enhance the development of a more positive perception (Leandro and Jay-Robert 2019, Kawahara et al. 2021, Vlasák‐Drücker et al. 2022). Negative perspectives alongside the lack of taxonomic knowledge by the public can be altered through educational tools and awareness raising campaigns (Lemelin et al. 2017, Weeks and Oseto 2018, Kawahara et al. 2021, Vlasák‐Drücker et al. 2022). The “Ants of Cyprus” website aims to disseminate all available scientific knowledge of the island’s ants providing species profiles for all known species. Some information are more relevant to the scientific community, for example, occurrence records as .xlsx files although visualised data, i.e. distribution maps and photographic material, can better help the public understand the biodiversity and spread of species. Furthermore, upon communication with education professionals, informational sources and educational material on taxonomy and morphology, alien species and citizen-science initiatives are presented in both Greek and English. Additionally, the “About ants” section is devoted to disseminating knowledge and scientific research on the morphology, species interrelationships, ecosystem functions and lifestyle of ants. Such material can be used during school field trips and biology classes when discussing the role of arthropods or insects and the biodiversity of Cyprus. This website will be further promoted to the public through Social Media. The website will be also promoted in and its data will be made available to established international repositories, such as AntWiki and AntMaps according to best practices' recommendation to scholarly publishing biodiversity data of Penev et al. (2017).

### Taxonomy

The “Ants of Cyprus” website offers an up-to-date, dynamic checklist of the myrmecofauna of Cyprus including statistics on the number of genera, species, endemic and alien taxa (see Statistics and Checklist page). These data are particularly important in assessing the overall biodiversity of ants on the island, as well as the relative percentages of endemic and alien species. Based on the latest published data, the island hosts 76 species of ants, including 17 endemic (22.37%) and 17 alien species (22.37%). This shows that Cyprus is an important biodiversity hotspot and endemism centre of ants, which has also been revealed in global scale analyses (Kass et al. 2022). However, the high percentage of alien species is also alarming. Nevertheless, correlating the island’s ant biodiversity with that of neighbouring Crete, an island of similar area that hosts more than 100 species (Salata et al. 2020, Borowiec and Salata 2022, Borowiec et al. 2023, Schifani et al. 2024), it is evident that the myrmecofauna of Cyprus is still largely under-sampled. Data on new records and species, detected during the course of the Darwin Fellowship (DPLUS200), published in scientific literature and discovered by citizen scientists (e.g. through iNaturalist or citizen-science initiatives) will be made available online as soon as these are published. Furthermore, the provision of photographic material from both own and online sources can enhance the aesthetic appeal of the website to non-taxonomists, as well as taxonomic investigations from specialists. The latter is further strengthened by the provision of sources regarding identification keys on the ants of Cyprus.

### Ecological data

On each species profile, a corresponding *.xlsx file is available, providing all published occurrence records. Data rows on each file are extracted from the published ant records dataset (Demetriou et al. 2024). Such data can be potentially utilised in analyses, such as species distribution modelling under climate change scenarios or potential impacts of land-use change. In addition, data on species interrelationships such as myrmecophiles, slave-making and slave species, predators, and parasitoids are given for each species and are databased for the whole island (in Media and Keys) in the hope of constructing ecological networks between ants and other species of the island. When used collectively, all occurrence records reveal under-sampled or unsampled areas where research is necessary.

### Conservation value

Following the application of the IUCN guideliness on reporting and assessment of species on a regional level, we provide data on the occurrence of species within protected areas, i.e. the NATURA2000 network, their AOO, EOO, lower and upper altitudinal limits, as well as relative information regarding their habitats. These can be used in future assessments on the extinction risk of Cypriot ants, as no species have been evaluated. Nevertheless, these data also provide valuable information on the biodiversity hosted by protected areas and can better help us map and evaluate the spread of alien and alien invasive species, such as the little fire ant *Wasmanniaauropunctata* and *Trichomyrmexdestructor* (Demetriou et al. 2023). Regarding alien species, the future application of EICAT and SEICAT impact assessment protocols is also needed in order to guide management practices.

## References

[B12223695] Angelidou Ιoanna, Demetriou Jakovos, Christou Maria, Koutsoukos Evangelos, Kazilas Christos, Georgiades Pantelis, Kalaentzis Konstantinos, Κontodimas Dimitris C., Groom Quentin, Roy Helen E., Martinou Angeliki F. (2022). Establishment and spread of the invasive ladybird *Harmoniaaxyridis* (Coleoptera: Coccinellidae) in Greece: based on contributions from citizen scientists. Biological Invasions.

[B12223711] AntWeb AntWeb Version 8.95.1. California Academy of Science. https://www.antweb.org.

[B12223719] Avtaeva T, Skripchinsky A, Brygadyrenko V (2020). Changes in the range of *Pterostichusmelas* and *P.fornicates* (Coleoptera, Carabidae) on the basis of climatic modeling. Baltic Journal of Coleopterology.

[B12223728] Bacher Sven, Blackburn Tim M., Essl Franz, Genovesi Piero, Heikkilä Jaakko, Jeschke Jonathan M., Jones Glyn, Keller Reuben, Kenis Marc, Kueffer Christoph, Martinou Angeliki F., Nentwig Wolfgang, Pergl Jan, Pyšek Petr, Rabitsch Wolfgang, Richardson David M., Roy Helen E., Saul Wolf‐Christian, Scalera Riccardo, Vilà Montserrat, Wilson John R. U., Kumschick Sabrina (2017). Socio‐economic impact classification of alien taxa (SEICAT). Methods in Ecology and Evolution.

[B12223755] Bachman Steven, Moat Justin, Hill Andrew, de la Torre Javier, Scott Ben (2011). Supporting Red List threat assessments with GeoCAT: geospatial conservation assessment tool. ZooKeys.

[B12223765] Beibl J., Buschinger A., Foitzik S., Heinze J. (2007). Phylogeny and phylogeography of the Mediterranean species of the parasitic ant genus Chalepoxenus and its *Temnothorax* hosts. Insectes Sociaux.

[B12223774] Blackburn Tim M., Essl Franz, Evans Thomas, Hulme Philip E., Jeschke Jonathan M., Kühn Ingolf, Kumschick Sabrina, Marková Zuzana, Mrugała Agata, Nentwig Wolfgang, Pergl Jan, Pyšek Petr, Rabitsch Wolfgang, Ricciardi Anthony, Richardson David M., Sendek Agnieszka, Vilà Montserrat, Wilson John R. U., Winter Marten, Genovesi Piero, Bacher Sven (2014). A unified classification of alien species based on the magnitude of their environmental impacts. PLOS Biology.

[B12223800] Bolton B. An online catalog of the ants of the world. https://antcat.org.

[B12223808] Borges P. A.V., Gabriel R., Arroz A. M., Costa A., Cunha R. T., Silva L., Mendonça E., Martins A. M.F., Reis F., Cardoso P. (2010). The Azorean Biodiversity Portal: An internet database for regional biodiversity outreach. Systematics and Biodiversity.

[B12223841] Borowiec L., Salata S. (2022). A monographic review of ants of Greece (Hymenoptera: Formicidae). Vol. 1. Introduction and review of all subfamilies except the subfamily Myrmicinae.

[B12223823] Borowiec Lech, Lapeva-Gjonova Albena, Salata Sebastian (2023). Second record of *Monomoriumexiguum* Forel, 1894 (Hymenoptera: Formicidae) from Europe. Annals of the Upper Silesian Museum of Bytom Entomology.

[B12223849] Buchelos C. T. (2012). Interpretative dictionary of entomological terms.

[B12223857] Buschinger A., Douwes P. (1993). Socially parasitic ants of Greece. Biologia Gallo-Hellenica.

[B12223866] Castañeda Saul, Botello Francisco, Sánchez-Cordero Víctor, Sarkar Sahotra (2019). Spatio-temporal distribution of monarch butterflies along their migratory route. Frontiers in Ecology and Evolution.

[B12223875] Claessens J,, Seifert B. (2017). Significant ant pollination in two orchid species in the Alps as adaptation to the climate of the alpine zone?. Tuexenia.

[B12223913] Group Darwin Core Maintenance (2021). Establishment means controlled vocabulary list of terms. Biodiversity Information Standards (TDWG). http://rs.tdwg.org/dwc/doc/em/2021-09-01.

[B12223884] Davranoglou Leonidas-Romanos, Karaouzas Ioannis (2021). Further distributional records of *Lethoceruspatruelis* (Stål, 1854) (Heteroptera: Belostomatidae) in Greece. Ecologica Montenegrina.

[B12223893] De Almeida Tania, Blight Olivier, Mesléard François, Bulot Adeline, Provost Erick, Dutoit Thierry (2020). Harvester ants as ecological engineers for Mediterranean grassland restoration: Impacts on soil and vegetation. Biological Conservation.

[B12223904] Del Toro Israel, Ribbons Relena R., Pelini Shannon L. (2012). The little things that run the world revisited: a review of ant-mediated ecosystem services and disservices (Hymenoptera: Formicidae). Myrmecological News.

[B12223935] Demetriou Jakovos, Georgiadis Christos, Roy Helen Elizabeth, Martinou Angeliki, Borowiec Lech, Salata Sebastian (2022). One of the world’s worst invasive alien species *Wasmanniaauropunctata* (Hymenoptera: Formicidae) Detected in Cyprus. Sociobiology.

[B12223921] Demetriou Jakovos, Georgiadis Christos, Martinou Angeliki F., Roy Helen E., Wetterer James K., Borowiec Lech, Economo Evan P., Triantis Kostas A., Salata Sebastian (2023). Running rampant: the alien ants (Hymenoptera, Formicidae) of Cyprus. NeoBiota.

[B12223946] Demetriou J., Georgiadis C., Salata S., Borowiec L., Dillen M., Groom Q., Economo E. P., Roy H. E., Martinou A. F. (2024). Published ant (Hymenoptera: Formicidae) records from the island of Cyprus.

[B12223959] Donaldson Michael R., Burnett Nicholas J., Braun Douglas C., Suski Cory D., Hinch Scott G., Cooke Steven J., Kerr Jeremy T. (2017). Taxonomic bias and international biodiversity conservation research. FACETS.

[B12223971] Dunn R. R. (2005). Modern insect extinctions, the neglected majority. Conservation Biology.

[B12223980] Edwards M., Varnava A., Stavrinides M., John E., Sparrow D. J., John E. (2016). ) An introduction to the wildlife of Cyprus.

[B12224020] Emery C. (1909). Beiträge zur Monographie der Formiciden des paläarktischen Faunengebietes. (Hym.). Deutsche Entomologische Zeitschrift.

[B12224029] Emery C. (1910). Beiträge zur Monographie der Formiciden des paläarktischen Faunengebietes. (Hym.). Deutsche Entomologische Zeitschrift.

[B12224038] Folgarait Patricia J. (1998). Ant biodiversity and its relationship to ecosystem functioning: a review. Biodiversity and Conservation.

[B12224047] Georghiou G. P. (1977). The insects and mites of Cyprus. With emphasis on species of economic importance to agriculture, forestry, man and domestic animals.

[B12224055] Goula Marta, Sesma José-Manuel, Vivas Luis (2013). Photosharing websites may improve Hemiptera biodiversity knowledge and conservation. ZooKeys.

[B12224064] Gruber Monica A. M., Santoro Davide, Cooling Meghan, Lester Philip J., Hoffmann Benjamin D., Boser Christina, Lach Lori (2022). A global review of socioeconomic and environmental impacts of ants reveals new insights for risk assessment. Ecological Applications.

[B12224076] Guénard B., Weiser M., Gomez K., Narula N., Economo E. P. (2017). The Global Ant Biodiversity Informatics (GABI) database: a synthesis of ant species geographic distributions. Myrmecological News.

[B12224095] Hand R., Hadjikyriakou G. N., Christodoulou C. S. Flora of Cyprus – a dynamic checklist. http://www.flora-of-cyprus.eu.

[B12224103] Ionescu-Hirsch A. (2009). An annotated list of *Camponotus* of Israel (Hymenoptera: Formicidae), with a key and descriptions of new species. Israel Journal of Entomology.

[B12224112] Janicki Julia, Narula Nitish, Ziegler Matt, Guénard Benoit, Economo Evan P. (2016). Visualizing and interacting with large-volume biodiversity data using client–server web-mapping applications: The design and implementation of antmaps.org. Ecological Informatics.

[B12224122] Jones Clive G., Lawton John H., Shachak Moshe (1997). Positive and negative effects of organisms as physical ecosystem engineers. Ecology.

[B12224139] Jouquet Pascal, Dauber Jens, Lagerlöf Jan, Lavelle Patrick, Lepage Michel (2006). Soil invertebrates as ecosystem engineers: Intended and accidental effects on soil and feedback loops. Applied Soil Ecology.

[B12224157] Kalaentzis Konstantinos, Kazilas Christos, Demetriou Jakovos, Koutsoukos Evangelos, Avtzis Dimitrios N., Georgiadis Christos (2021). Alientoma, a dynamic database for alien insects in Greece and its use by citizen scientists in mapping alien species. Insects.

[B12224168] Kass Jamie M., Guénard Benoit, Dudley Kenneth L., Jenkins Clinton N., Azuma Fumika, Fisher Brian L., Parr Catherine L., Gibb Heloise, Longino John T., Ward Philip S., Chao Anne, Lubertazzi David, Weiser Michael, Jetz Walter, Guralnick Robert, Blatrix Rumsaïs, Lauriers James Des, Donoso David A., Georgiadis Christos, Gomez Kiko, Hawkes Peter G., Johnson Robert A., Lattke John E., MacGown Joe A., Mackay William, Robson Simon, Sanders Nathan J., Dunn Robert R., Economo Evan P. (2022). The global distribution of known and undiscovered ant biodiversity. Science Advances.

[B12224204] Kawahara Akito Y., Reeves Lawrence E., Barber Jesse R., Black Scott H. (2021). Eight simple actions that individuals can take to save insects from global declines. Proceedings of the National Academy of Sciences.

[B12224217] Klotz J. H., Mangold J. R., Vail K. M., Davis Jr L. R., Patterson R. S. (1995). A survey of the urban pest ants (Hymenoptera: Formicidae) of peninsular Florida. Florida Entomologist.

[B12224227] Koutsoukos Evangelos, Demetriou Jakovos, Georgiadis Christos, Mitroiu Mircea-Dan, Compton Stephen, Martinou Angeliki (2024). Highlighting overlooked biodiversity through online platforms: The “Chalcid Wasps of Cyprus” website. Biodiversity Data Journal.

[B12224238] Krapf Patrick (2023). Contribution of the public to the modelling of the distributions of species: Occurrence and current and potential distribution of the ant *Manicarubida* (Hymenoptera: Formicidae). European Journal of Entomology.

[B12224247] Lard C. F., Schmidt J., Morris B., Estes L., Ryan C., Bergquist D. (2006). An economic impact of imported fire ants in the United States of America.

[B12224257] Leandro Camila, Jay-Robert Pierre (2019). Perceptions and representations of animal diversity: Where did the insects go?. Biological Conservation.

[B12224280] Lee C. Y. (2002). Tropical household ants – pest status, species diversity, foraging behavior and baiting studies.

[B12224289] Lemelin R. H., Dampier J., Harper R., Bowles R., Balika D. (2017). Perceptions of insects: a visual analysis. Society and Animals.

[B12224306] Lengyel Szabolcs, Gove Aaron D., Latimer Andrew M., Majer Jonathan D., Dunn Robert R. (2010). Convergent evolution of seed dispersal by ants, and phylogeny and biogeography in flowering plants: A global survey. Perspectives in Plant Ecology, Evolution and Systematics.

[B12224316] Lugo David, Suárez Daniel, Martín Sonia, Afonso Óscar, Martín Alicia, Ruiz Carlos (2023). First record of *Leptoglossusoccidentalis* Heidemann, 1910 (Hemiptera, Coreidae) in the Canary Islands, a novel pine pest detected through citizen science in an oceanic archipelago. Biodiversity Data Journal.

[B12224327] Luke Sarah H., Roy Helen E., Thomas Chris D., Tilley Luke A. N., Ward Simon, Watt Allan, Carnaghi Manuela, Jaworski Coline C., Tercel Maximillian P. T. G., Woodrow Charlie, Aown Susmita, Banfield‐Zanin Jennifer A., Barnsley Sarah L., Berger Iris, Brown Mark J. F., Bull James C., Campbell Heather, Carter Ruth A. B., Charalambous Magda, Cole Lorna J., Ebejer Martin J., Farrow Rachel A., Fartyal Rajendra S., Grace Miriam, Highet Fiona, Hill Jane K., Hood Amelia S. C., Kent Eleanor S., Krell Frank‐Thorsten, Leather Simon R., Leybourne Daniel J., Littlewood Nick A., Lyons Ashley, Matthews Graham, Mc Namara Louise, Menéndez Rosa, Merrett Peter, Mohammed Sajidha, Murchie Archie K., Noble Michael, Paiva Maria‐Rosa, Pannell Michael J., Phon Chooi‐Khim, Port Gordon, Powell Charlotte, Rosell Stewart, Sconce Francisca, Shortall Chris R., Slade Eleanor M., Sutherland Jamie P., Weir Jamie C., Williams Christopher D., Zielonka Natalia B., Dicks Lynn V. (2023). Grand challenges in entomology: Priorities for action in the coming decades. Insect Conservation and Diversity.

[B12224386] McCormack G. Cook Islands biodiversity database. Cook Islands Natural Heritage Trust, Rarotonga. http://cookislands.bishopmuseum.org.

[B12224394] Mesibov Robert (2019). An archived taxonomic website: Millipedes of Australia. Biodiversity Data Journal.

[B12224403] Montgomery M P, Vanderwoude C, Lintermans M, Lynch A J Jasmyn (2022). The little fire ant (Hymenoptera: Formicidae): A global perspective. Annals of the Entomological Society of America.

[B12224412] Moreira Denise D. O., Morais Verônica de, Vieira-da-Motta Olney, Campos-Farinha Ana E. de C., Tonhasca Jr. Athayde (2005). Ants as carriers of antibiotic-resistant bacteria in hospitals. Neotropical Entomology.

[B12224422] Myers Norman, Mittermeier Russell A., Mittermeier Cristina G., da Fonseca Gustavo A. B., Kent Jennifer (2000). Biodiversity hotspots for conservation priorities. Nature.

[B12224086] Oberhauser Karen, Guiney Margaret (2009). Insects as flagship conservation species. Terrestrial Arthropod Reviews.

[B12383189] Parker Joseph, Kronauer Daniel J. C. (2021). How ants shape biodiversity. Current Biology.

[B12224432] Penev Lyubomir, Mietchen Daniel, Chavan Vishwas, Hagedorn Gregor, Smith Vincent, Shotton David, Ó Tuama Éamonn, Senderov Viktor, Georgiev Teodor, Stoev Pavel, Groom Quentin, Remsen David, Edmunds Scott (2017). Strategies and guidelines for scholarly publishing of biodiversity data. Research Ideas and Outcomes.

[B12224450] Roy Helen E., Pauchard Aníbal, Stoett Peter, Renard Truong Tanara, Lipinskaya Tatsiana, Vicente Joana R. (2024). IPBES Invasive Alien Species Assessment: Chapter 1. Introducing biological invasions and the IPBES thematic assessment of invasive alien species and their control. Zenodo.

[B12224461] Ruzzier Enrico, Menchetti Mattia, Bortolotti Laura, Selis Marco, Monterastelli Elisa, Forbicioni Leonardo (2020). Updated distribution of the invasive *Megachilesculpturalis* (Hymenoptera: Megachilidae) in Italy and its first record on a Mediterranean island. Biodiversity Data Journal.

[B12224472] Salata S., Borowiec L. (2015). A taxonomic revision of the genus *Oxyopomyrmex* André, 1881 (Hymenoptera: Formicidae). Zootaxa.

[B12224481] Salata Sebastian, Borowiec Lech (2015). Redescription of *Crematogastercypria* Santschi, 1930, new status, with description of two new related species from Greece and Turkey (Hymenoptera, Formicidae). ZooKeys.

[B12224535] Salata S., Georgiadis C., Borowiec L. (2019). Invasive ant species (Hymenoptera: Formicidae) of Greece and Cyprus. North-Western Journal of Zoology.

[B12224490] Salata S., Borowiec L., Trichas A. (2020). Review of ants (Hymenoptera: Formicidae) of Crete, with keys to species determination and zoogeographical remarks. Monographs of the Upper Silesian Museum.

[B12224499] Salata Sebastian, Demetriou Jakovos, Georgiadis Christos, Borowiec Lech (2023). *Camponotus* Mayr, 1861 (Hymenoptera: Formicidae) of Cyprus: generic synopsis and description of a new species. Asian Myrmecology, (Asian Myrmecol.).

[B12224508] Salata S., Demetriou J., Georgiadis C., Borowiec L. (2023). The ant genus *Cataglyphis* Förster (Hymenoptera: Formicidae) in Cyprus. Zootaxa.

[B12224517] Salata Sebastian, Demetriou Jakovos, Georgiadis Christos, Borowiec Lech (2023). The genus *Messor* Forel, 1890 (Hymenoptera: Formicidae) in Cyprus. Annales Zoologici.

[B12224526] Salata S., Demetriou J., Georgiadis C., Borowiec L. (2024). The genus *Temnothorax* Mayr, 1861 (Hymenoptera: Formicidae) in Cyprus. Zootaxa.

[B12224544] Santschi F. (1930). Trois notes myrmécologiques. Bulletin et annales de la Société royal e Belge d'entomologie.

[B12224553] Santschi F (1934). Contribution aus Solenopsis paléarctiques. Revue suisse de zoologie.

[B12224562] Santschi F. (1937). Contribution à l'étude des *Crematogaster* paléarctiques. Mémoires de la Société vaudoise des sciences naturelles.

[B12224571] Schifani Enrico, Georgiadis Christos, Menchetti Mattia (2024). *Cardiocondylaobscurior*, a new alien ant in Crete (Hymenoptera, Formicidae). Biogeographia – The Journal of Integrative Biogeography.

[B12224580] Schultheiss Patrick, Nooten Sabine S., Wang Runxi, Wong Mark K. L., Brassard François, Guénard Benoit (2022). The abundance, biomass, and distribution of ants on Earth. Proceedings of the National Academy of Sciences.

[B12224591] Seifert Bernhard (2003). The ant genus *Cardiocondyla* (Insecta: Hymenoptera: Formicidae) - a taxonomic revision of the *C.elegans*, *C.bulgarica*, *C.batesii*, *C.nuda*, *C.shuckardi*, *C.stambuloffii*, *C.wroughtonii*, *C.emeryi*, and *C.minutior* species groups. Annalen des Naturhistorischen Museums in Wien, Serie B, Botanik und Zoologie (Ann. Naturhist. Mus. Wien).

[B12224618] Seifert B., Schultz R. (2009). A taxonomic revision of the *Formicarufibarbis* Fabricius, 1793 group (Hymenoptera: Formicidae). Myrmecological News.

[B12224600] Seifert B. (2020). A taxonomic revision of the Palaearctic members of the subgenus Lasius s.str. (Hymenoptera, Formicidae). Soil Organisms.

[B12224627] Serge M. M.P., Giovani E. T., Mony R. (2019). Household and home garden infesting arthropods (ants and myriapods) in the city of Yaoundé, Cameroon. Journal of Entomology and Zoology Studies.

[B12224636] Skvarla Michael J, Fisher J Ray (2023). Online community photo-sharing in entomology: a large-scale review with suggestions on best practices. Annals of the Entomological Society of America.

[B12224645] Tercel Maximillian P. T. G., Cuff Jordan P., Symondson William O. C., Vaughan Ian P. (2023). Non-native ants drive dramatic declines in animal community diversity: A meta-analysis. Insect Conservation and Diversity.

[B12224654] Troudet Julien, Grandcolas Philippe, Blin Amandine, Vignes-Lebbe Régine, Legendre Frédéric (2017). Taxonomic bias in biodiversity data and societal preferences. Scientific Reports.

[B12224664] Vlasák‐Drücker Jasmin, Eylering Annike, Drews Jasmin, Hillmer Gesa, Carvalho Hilje Vera, Fiebelkorn Florian (2022). Free word association analysis of Germans' attitudes toward insects. Conservation Science and Practice.

[B12224675] Weeks Faith, Oseto Christian (2018). Interest in Insects: The Role of Entomology in Environmental Education. Insects.

[B12224684] Wetterer James K. (2009). Worldwide spread of the destroyer ant, *Monomoriumdestructor* (Hymenoptera: Formicidae). Myrmecological News.

[B12224693] Wetterer J. K., Porter S. D. (2003). The little fire ant, *Wasmanniaauropunctata*: distribution, impact, and control. Sociobiology.

[B12224702] Wieczorek John, Bloom David, Guralnick Robert, Blum Stan, Döring Markus, Giovanni Renato, Robertson Tim, Vieglais David (2012). Darwin Core: An Evolving Community-Developed Biodiversity Data Standard. PLOS One.

[B12224715] Wilson Joseph S., Pan Aaron D., General David Emmanuel M., Koch Jonathan B. (2020). More eyes on the prize: an observation of a very rare, threatened species of Philippine Bumble bee, *Bombusirisanensis*, on iNaturalist and the importance of citizen science in conservation biology. Journal of Insect Conservation.

[B12224724] Zina Vera, Branco Manuela, Franco José Carlos (2020). Impact of the invasive Argentine ant in *Citrus* agroecosystems: Effects on the diversity and frequency of native ant species foraging on tree canopy. Insects.

[B12224741] Zotos Savvas, Stamatiou Marilena, Vogiatzakis Ioannis (2023). The Cyprus Herp Atlas: An initiative for systematic recording of amphibian and reptile occurrences in Cyprus. Biodiversity Data Journal.

